# Fertility discussions and concerns in childhood cancer survivors, a systematic review for updated practice

**DOI:** 10.1002/cam4.5339

**Published:** 2022-10-12

**Authors:** Karima El Alaoui‐Lasmaili, Phi Linh Nguyen‐Thi, Nadine Demogeot, Joëlle Lighezzolo‐Alnot, Marie José Gross, Ludovic Mansuy, Pascal Chastagner, Isabelle Koscinski

**Affiliations:** ^1^ Interpsy Laboratory (UR4432) University of Lorraine Nancy France; ^2^ Unité d'évaluation médicale, Unité de Méthodologie Data management et Statistique – UMDS, CHRU de Nancy; ^3^ Psychotherapeutic Center of Nancy Laxou France; ^4^ Department of Pediatric Hematology and Oncology University Hospital of Nancy Vandœuvre‐lès‐Nancy France; ^5^ Laboratory of Biology of Reproduction‐CECOS Lorraine University Hospital of Nancy Nancy France; ^6^ INSERM U1256, NGERE, Université de Lorraine Nancy France

**Keywords:** adolescent, cancer survivors, child, fertility preservation, infertility, psychology

## Abstract

**Purpose:**

To provide ways to improve the clinical practice of fertility preservation (FP) for children, adolescents, and young adults (AYA) with cancer.

**Design:**

A systematic research of online databases was undertaken in March 2020 following the PRISMA criteria, including Medline and Web of Science.

**Results:**

Fifty‐nine articles were included. Surveys, interviews, and focus groups were used to collect data from patients, parents, and health care providers (HCPs). Four themes worth exploring emerged: (a) what do patients and professionals think of and know about FP? (b) what makes the fertility discussion happen or not? (c) what, retrospectively, led to FP being pursued or not? and (d) how do patients and HCPs feel about fertility issues?

**Conclusion:**

A minority of AYAs preserve their fertility (banking assay for 45% of boys and 23% of girls). Yet fertility concerns have a significant impact on the quality of life of young cancer survivors. Although recommendations and guidelines regarding FP are available internationally, there are no specific guidelines as to how to conduct fertility counseling for children and adolescents. Some barriers are not removable, such as a poor prognosis of an obvious severe disease, time constraints for starting treatment, and cultural and religious beliefs. In response to aspects hindering patients and families to be receptive to any discussion at the time of diagnosis, psychological support could reduce the level of emotional distress and help restore a degree of open‐mindedness to open a window for discussion. Moreover, as the lack of knowledge of professionals about fertility is frequently pointed out as a limiting factor for fertility discussion, reinforcing professional training regarding FP could be proposed to promote fertility discussion and eventually referral for FP.

## Graphical abstract

This review studies the mechanisms leading to fertility preservation with a focus on the behavior and psychosocial issues surrounding FP in young cancer survivors, their parents and health care professionals.

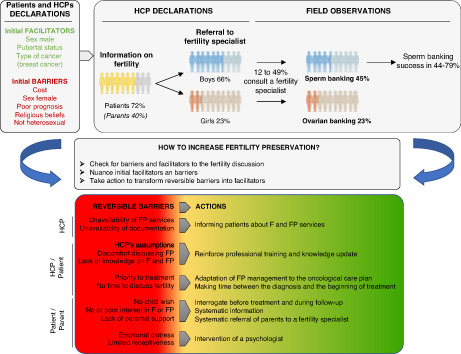

## 1 INTRODUCTION

The prognosis of children with cancer has improved considerably for more than a decade, with one in 500 adults now a Childhood Cancer Survivor (CCS) and expressing concerns about their health, body image, relationships, and the desire to have children.^1^ Cancer treatments such as surgery, chemotherapy, and radiotherapy can alter fertility^2^, ^3^ and therefore international focus groups,^4^ PanCareLIFE,^5^ and national health services^6^, ^7^ recommend to informing patients and their families about the risk of infertility and to propose fertility preservation if possible. However, there are major discrepancies between the recommendations and the reality of the practice.^8^, ^9^ Most of the time, cancer diagnosis represents such a life‐threatening emergency that many children, adolescents, and some young adults go through cancer treatment without having been told about fertility issues, only discovering this later on when they face difficulties conceiving a child.

This Systematic Literature Review (SLR) studies the mechanisms leading to fertility preservation by gathering and analyzing articles that consider all the aspects of FP in prepubescent and pubescent patients to approach this matter in its entirety. We hence chose to compile the perspectives of patients, parents, and health care professionals (HCPs) to better decipher the complexity of the realities of the practice and focus on the behavior and psychosocial issues surrounding FP. To do so, this SLR will strive to answer four questions: (a) what do patients and professionals think and know about FP? (b) what makes the fertility discussion happen or not? (c) what, retrospectively, led to FP being pursued or not? and (d) how do patients and HCPs feel about fertility issues?

## 2 MATERIALS AND METHODS

### 2.1 Search strategy, inclusion, and exclusion criteria

**FIGURE 1:**
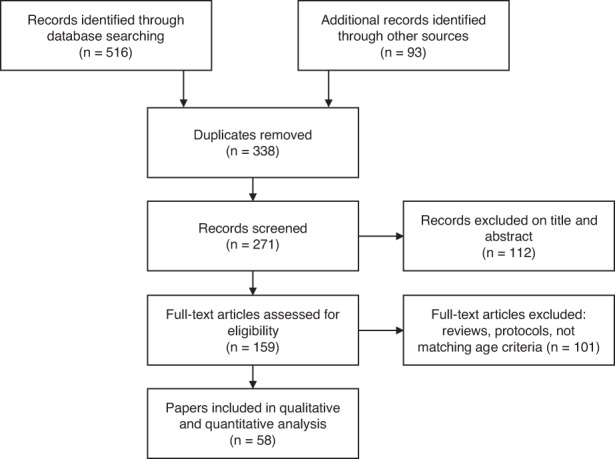
PRISMA flowchart for inclusion and exclusion of papers reporting on concerns, psychosocial or psychological needs in children or adolescents facing cancer and fertility issues.

A systematic search of online databases available at the University of Lorraine was undertaken in March 2020 following the Preferred Reporting Items for Systematic Reviews and Meta‐Analyses (PRISMA).^10^ This included Medline, Web of Science, PMC, Springer, Mary Ann Liebert, Nature Publishing Group, Elsevier, Oxford Journals, Taylor & Francis, Wiley, and Wolters Kluwer. The terms of the search were chosen to reflect cancer and fertility management and their psychological impact on patients treated during childhood and adolescence. The subject terms chosen were “cancer” and “fertility.” The articles were retrieved when the following terms were in the text of the article: “childhood” or “adolescence” and “concern” or “psychosocial” or “psychology.” A total of 609 articles were identified via the database searches and additional manual screening (Figure 1). After removal of duplicates (338 articles), 271 records were screened for the title and abstract (by a single reviewer), and the remaining 159 were read in full. The exclusion criteria were discussed with a second reviewer to determine the inclusion eligibility of the remaining articles. For instance, the papers related exclusively to adult patients diagnosed with cancer after the age of 25 were excluded because their concerns, social and family support and the representation of fertility are different.

The inclusion criteria were met when (a) the populations of interest were children and adolescents, whether it was their point of view, that of the parent’ or the physician/health care providers, (b) the study was conducted retrospectively and patients had finished their treatments, (c) it contained data on the fertility‐related concerns, distress, discussion, and practice, (d) the methodology was qualitative, quantitative, or both and based on surveys, interviews or focus groups. In total, 58 articles were included in this review.

### 2.2 Quality assessment

The quality of the articles was independently assessed by two researchers (K.E and I.K). The selection was based on the clinical relevance and validity of the methodology. Qualitative studies were evaluated using factors that accounted for relevance and validity^11^ that met the aims of this SLR. Studies judged as weak in terms of the methodology quality were withdrawn. As the quantitative studies included were mostly psychology‐oriented and non‐interventional, it did not seem appropriate to use complex grading systems devised for medical cohort studies.^12^

### 2.3 Age classification

The populations were sorted by the age reported in the inclusion criteria, representing children (0–13), adolescents and young adults (13–21), and young adults (21–25). When available, both “age at diagnosis” and “age at research” were reported. Due to the lack of consensus in defining age categories for children and adolescents with cancer, some studies overlapped between the two intervals (Table S1).

### 2.4 Article classification

After the initial identification of 609 articles, the 58 articles meeting the inclusion criteria and quality requirements proved to be rather heterogenous in terms of the research question, content, and methodology. Therefore, a systematic review of the literature was more appropriate than a meta‐analysis. A pilot study was undertaken on 10 random articles from the final 58 to identify the criteria to be analyzed. The following four themes emerged from this preliminary analysis: (a) patient and professional knowledge and opinion regarding fertility preservation, (b) facilitators and barriers to fertility discussion, (c) reasons for pursuing or forgoing fertility preservation, and (d) emotions and concerns related to potential infertility. This classification was validated by all the authors. Each article was assigned to at least one theme.

Facilitators and barriers were defined by personal, clinical, and institutional factors that were described in the original articles as reasons why the fertility discussion took place or not

## 3 RESULTS

Forty‐one of the 58 selected articles utilized quantitative methods, 11 utilized qualitative analyses, and 7 utilized mixed methods (Table 1). More specifically, the data were collected by means of a survey in 41 cases, by interviews in 7 cases, by both a survey and an interview in 6 cases, by the analysis of the content of the discussions in a focus group in 3 cases, and by both an interview and a focus group in 2 cases. The studies were published between 2007 and 2020 across 14 countries representing in North America (*n* = 39), Europe (*n* = 16), Australia (*n* = 5), and Asia (*n* = 1).

**TABLE 1:** Characteristics of the population and the methodologies of the 58 articles included. The articles are described in totality and within each category they were assigned to: “Experience of fertility issues”, “Concerns” (for “Patient's perceptions, emotions and concerns related to (potential) infertility”), “Facilitators and barriers to the fertility discussion”, and “reasons for pursuing or foregoing fertility preservation (FP)”.

	Total	Experience with fertility issues	Concerns	Facilitators and Barriers	Reasons for pursuing or foregoing FP
No. of articles	58	38	32	22	15
Study design					
Qualitative	11	7	10	1	1
Quantitative	40	28	18	19	11
Mixed‐Methods	7	3	4	2	3
Data‐collection method					
Survey	40	29	18	20	12
Interview	7	4	6	1	1
Focus group	3	1	3	—	—
Interview and focus group	2	2	1	—	—
Survey and interview	6	2	4	1	2
Study population					
Patient	34	22	25	6	8
Parent	2	2	1	1	1
HCP	12	7	‐	12	3
Patient and parent	8	5	6	1	2
Patient and HCP	1	1	—	1	—
Patient, Parent and HCP	1	1	—	1	1
Targeted population					
Prepubescent only	25	19	14	8	6
Pre and Postpubescent	58	38	32	22	15
Total number of patients included					
Total patients	**11,257**	**7748**	**7746**	**3816**	**3902**
Median	102	99	63	177	110
Mean	245	250	242	382	325
Interval	[8–2489]	[10–2489]	[8–2489]	[82–1169]	[23–2489]
Females	**7275**	**4897**	**5053**	**2486**	**1786**
Median	63	57	51	328	123
Mean	218	223	194	497	298
Interval	[6–1322]	[6–1322]	[6–1322]	[120–1169]	[19–1322]
Total number of patients included					
Males	**4055**	**2734**	**2576**	**1330**	**2116**
Median	52	52	32	146	120
Mean	115	130	107	148	235
Interval	[5–1167]	[5–1167]	[5–1167]	[82–283]	[28–1167]
Parents	**864**	**596**	**456**	**288**	**264**
Median	97	109	23	144	132
Mean	86	99	65	144	132
Interval	[7–202]	[10–202]	[7–202]	[144]	[120–144]
HCPs	**1195**	**603**	—	**1143**	**346**
Median	52	51	—	52	52
Mean	76	60	—	76,2	69
Interval	[9–209]	[9–209]	—	[9–209]	[9–209]
Age at research					
[0–13]	—	5	4	1	2
[13–21]	—	19	18	7	9
[21–25]	—	20	25	6	5
Age at diagnosis					
[0–13]	—	17	15	4	6
[13–21]	‐	26	22	7	10
[21–25]	‐	7	3	4	‐
Type of cancer					
Hematologic	4071	1800	1634	2550	657
Neurologic	310	286	222	69	127
Germ cell and gynecological	2546	1753	1928	1827	350
Sarcomas	262	414	331	88	198
Other	1407	1970	1813	207	1191
Country of study					
USA	34	20	16	9	7
Europe	17	12	8	7	4
Australia	6	3	5	2	1
Asia	1	—	—	1	—

### 3.1 Characteristics of the population included in the studies

Most of the studies surveyed only patients (*n* = 34), 12 surveyed HCPs, and 2 surveyed only parents of children or adolescents who had been treated for cancer. Eight articles studied both patients and parents, one included patients and HCPs, and one included patients, parents, and HCPs. Finally, 25 studies were related exclusively to prepubescent patients (Table 1).

The number of patients included in the studies ranged from 8 to 2489, with a mean of 244 patients. A mean number of 218 female (6–1322) and 115 male (5–1167) patients were included in the studies. Parents were included in 11 studies (mean = 86, range: 7–202). HCPs were included in 14 studies (mean = 76, range: 9–209).

The most common diagnoses were hematological cancers (47%), germ cell cancers, and gynecological cancers (30%). Neurological cancers and sarcomas represented 4% and 3%, respectively of the patients.

### 3.2 Patient and professional knowledge and opinion regarding fertility preservation

#### 3.2.1 Patients

Less than 20% (94/549) of the patients surveyed did not know their reproductive health status^13^, ^14^, ^15^, ^16^, ^17^, ^18^, ^19^, ^20^, ^21^ (Table S2), the others reported limited or uncertain knowledge, while some patients and parents expected normal fertility. However, 42% (1813/4419) of the patients surveyed remembered having been informed about the risk of potential infertility,^13^, ^16^, ^18^, ^20^, ^21^, ^22^, ^23^, ^24^, ^25^, ^26^, ^27^, ^28^, ^29^, ^30^, ^31^, ^32^, ^33^ and 49% (1079/2218) remembered having discussed FP options.^17^, ^18^, ^19^, ^20^, ^34^, ^35^, ^36^, ^37^, ^38^, ^39^ Twenty‐three percent of the patients (491/2139) were referred to a fertility specialist,^3^, ^19^, ^20^, ^30^, ^31^, ^36^, ^40^, ^41^, ^42^ and ultimately 21% of the surveyed patients underwent FP (645/3051).^1^, ^13^, ^14^, ^15^, ^17^, ^18^, ^20^, ^35^, ^36^, ^37^, ^38^, ^40^, ^42^, ^43^, ^44^, ^45^, ^46^

Only 12% of eligible pubescent girls underwent oocyte preservation (225/1797),^17^, ^20^, ^35^, ^36^, ^40^, ^41^ while 45% of boys partook in sperm banking (329/731).^1^, ^16^, ^17^, ^31^, ^35^, ^40^, ^42^, ^44^, ^47^ Ovarian tissue was preserved in 145/1267 cases (11%),^40^, ^41^ but there were no reports of testicular tissue preservation. Reports of embryos preservation were included (Table S2) and involved a third of these patients (505/1521)^30^, ^34^, ^48^ since three articles also included young adults. Use of hormonal therapy was reported in 4% (24/605) of the patients in four studies.^27^, ^32^, ^44^, ^48^ Shielding or ovarian transposition was reported in 14% (164/1169) of the patients in one cohort.^34^

Evaluation of fertility testing after cancer treatment was mentioned in only two studies,^16^, ^26^ with 45% (62/137) of the patients having been tested.

#### 3.2.2 HCPs

The papers on the outlook of HCPs either provided information regarding the practice^49^, ^50^, ^51^, ^52^, ^53^ or yielded tangible results^31^, ^40^, ^54^, ^55^ (Table S3). Seventy‐two percent of HCPs stated having discussed potential infertility with the patients^31^ and 40% with the parents of children under 18 years of age.^40^ In approximately half of the cases written information was provided.^30^, ^31^ FP options were presented before the beginning of treatment in 2/9 papers.^49^, ^50^ Only selected patients were referred to a fertility specialist: only pubescents,^51^ more frequently boys (66%) than girls (23%),^50^ and sometimes only when patients and parents expressed concerns regarding infertility.^51^ In practice, referral was observed in 12% to 49% of cases,^30^, ^40^, ^54^ which corresponded to the proportion of patients who underwent FP.^40^, ^54^ Overall, even a routine practice such as sperm banking, was proposed in 54% to 96% of pubescent patients,^31^, ^40^, ^50^, ^51^, ^52^, ^53^, ^54^, ^55^ with a very heterogeneous success rate (44% to 79%).^31^, ^40^, ^55^ Interestingly, Chong et al.^40^ pointed out the loss of patients throughout the course of the banking process, from 85/172 referred patients, 38/85 agreeing to have tissue collected, and ultimately 30/38 to successfully banking. For a new practice such as oocyte or germinal tissue preservation, the gap was even greater between patients interested in FP and the number of cryopreservations carried out.^49^, ^50^, ^51^, ^52^, ^53^, ^54^ Shielding and transposition^52^ as well as the use of GnRH were rarely reported.^52^, ^54^

### 3.3 Facilitators and barriers to the fertility discussion

**FIGURE 2:**
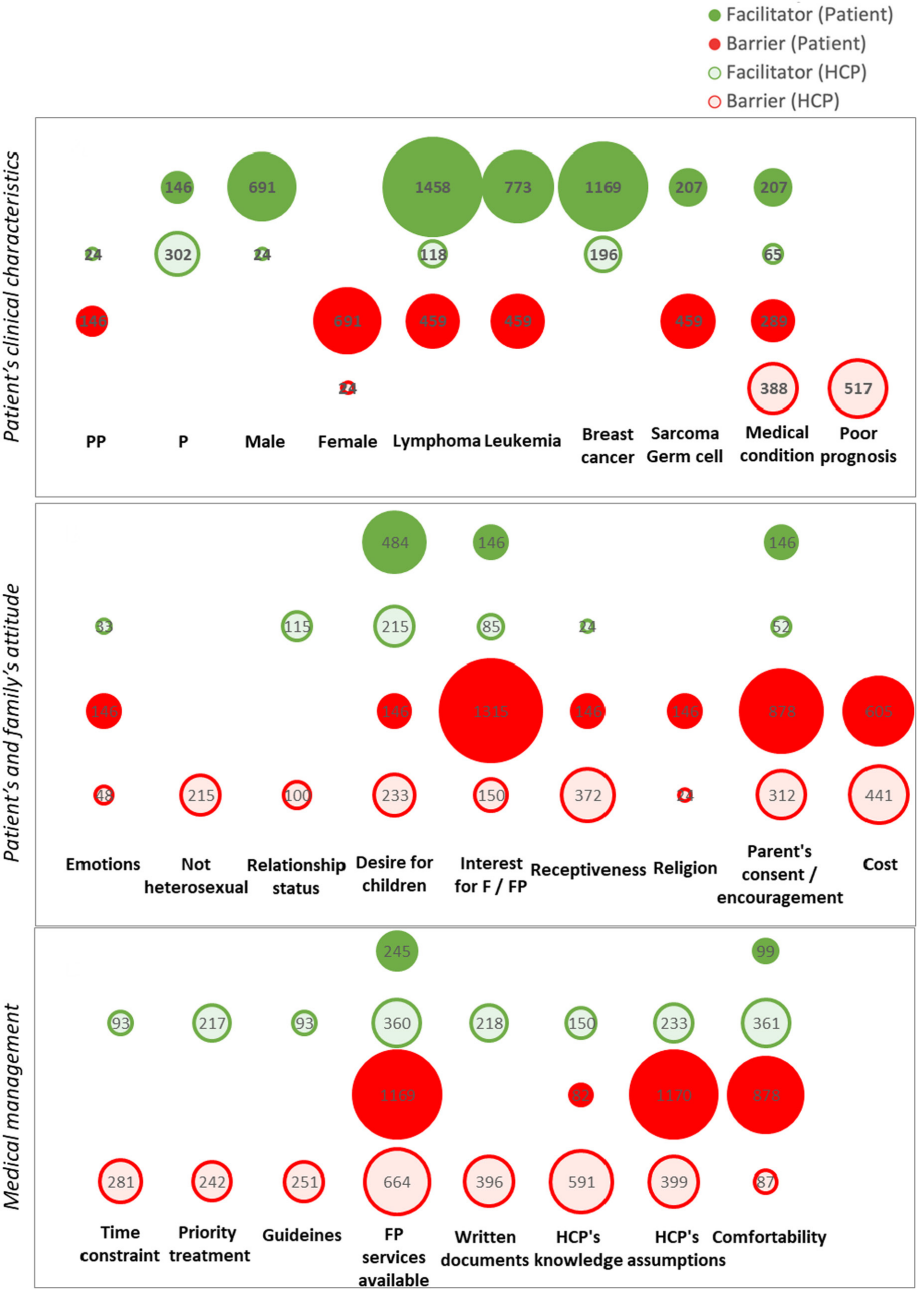
Facilitators and barriers to the fertility discussion. Parameters related to the patient's clinical characteristics, the patient's and family's attitude, and to the medical management were found to be facilitators (green circles) and/or barriers (red circles) to fertility discussion and are presented as the point of view of patients (filled circles) or HCPs (clear circles). The size of the circles represents the number of patients or HCPs indicating each factor as a facilitator or as a barrier in the survey.

Twenty‐seven parameters considered to be facilitators and/or barriers to fertility discussion were identified for patients and/or HCPs (Figure 2, Table S4). They are related to the patient's clinical characteristics, the patient's and the family's attitude, and medical management.

Pubertal status,^40^, ^49^, ^50^, ^55^, ^56^ male gender^29^, ^48^, ^49^ and breast cancer^34^, ^57^ appeared as exclusive facilitators, while female gender,^29^, ^48^, ^49^ poor life expectancy,^50^, ^57^, ^58^, ^59^ non‐heterosexual orientation,^58^, ^59^ religion,^49^, ^56^ and the cost of FP^28^, ^40^, ^49^, ^50^, ^56^, ^57^, ^59^ were exclusive barriers. Relationship status^58^, ^59^ was a barrier exclusively for HCPs, especially for unmarried patients.

Apart from breast cancer which was borderline data considering the focus of this review on CCS, there was no link between the type of cancer and the fertility discussion.

Some factors stood out as facilitators as well as barriers for both patients and HCPs: the general health status,^29^, ^40^, ^49^, ^50^, ^51^, ^59^, ^60^, ^61^ the desire to have children,^48^, ^49^, ^50^, ^56^, ^58^, ^59^ the parent's support to discuss fertility and perform FP,^35^, ^40^, ^49^, ^50^, ^52^, ^55^, ^56^, ^62^ the availability of FP services,^31^, ^34^, ^40^, ^49^, ^51^, ^53^, ^55^, ^57^, ^58^, ^59^, ^61^, ^63^ the HCP's assumptions,^35^, ^40^, ^49^, ^50^, ^55^, ^56^, ^57^, ^58^, ^59^ and comfortability to discussing fertility.^31^, ^35^, ^40^, ^49^, ^50^, ^59^, ^61^

The patient's attitude could have been the source of additional barriers to fertility discussion: emotional distress^40^, ^49^, ^54^, ^56^ and limited receptiveness,^40^, ^49^, ^50^, ^54^, ^56^, ^58^ not wanting children,^50^, ^56^ no or little interest in fertility and FP,^34^, ^54^, ^56^, ^57^, ^61^ and religious views.^50^, ^56^

In HCP's attitude, barriers were found in: the lack of time to discuss fertility,^40^, ^50^, ^54^, ^57^, ^61^ the priority given to starting treatment,^49^, ^53^, ^57^, ^59^ and the lack of documentation for patients.^49^, ^53^, ^58^, ^61^, ^63^ Moreover, they had significant reservations regarding their lack of knowledge about fertility issues, preservation options, and the efficacy of Assisted Reproductive Technologies (ART).^50^, ^51^, ^59^, ^60^, ^61^, ^63^

### 3.4 Reasons for pursuing or foregoing FP

**FIGURE 3:**
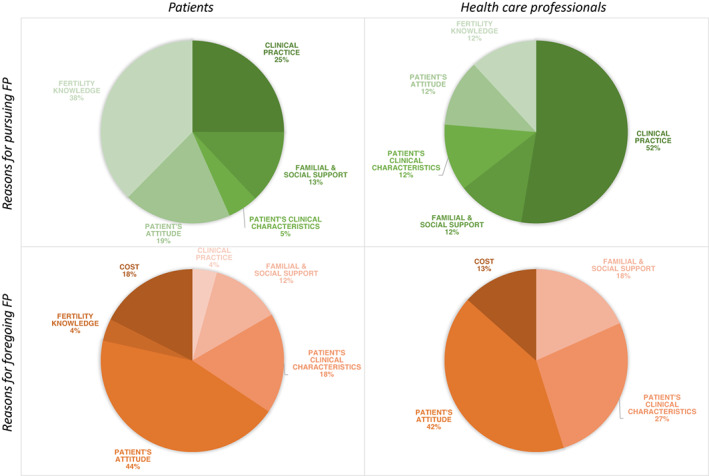
Reasons for pursuing or foregoing fertility preservation. The reasons for pursuing or foregoing fertility preservation (FP) were sorted into six categories and presented as the point of view of patients and health care professionals.

The reasons for pursuing or foregoing FP were sorted into six categories and analyzed from the point of view of patients and HCPs (Figure 3, Table S5): (a) Clinical practice that regrouped factors related to the physician, the FP consultation and facilities, (b) familial and social support, (c) patient's clinical characteristics, (d) patient's attitude, (e) knowledge regarding fertility and fertility preservation options, and (f) cost.

For patients, the most important reason to pursue FP was their knowledge of fertility (38% of the cases), especially when endorsing the benefits of banking.^38^, ^55^, ^56^ The second most important factor was the clinical practice (25% of the cases), when the physician encouraged the parents^49^ and the patients,^25^, ^55^ and initiated consultation with a fertility specialist.^30^, ^31^, ^43^, ^44^, ^55^, ^56^ The patient's attitude represented 19% of the reasons to pursue FP (own perception of FP,^25^, ^55^ desire to have children, and interest in being a parent^24^, ^25^, ^34^). This was followed by the familial and social support (13% of the cases)^25^, ^43^, ^55^, ^56^ and the clinical characteristics such as the stage of pubertal development (5% of the cases).^55^, ^56^

In contrast, for HCPs, fertility knowledge,^38^, ^55^, ^56^ patient's attitude,^25^, ^55^ patient's clinical characteristics,^55^, ^56^ and support^25^, ^55^, ^56^ together weighed less than the clinical practice.^25^, ^30^, ^31^, ^43^, ^44^, ^49^, ^55^, ^56^

Patients and HCPs agreed that the main factors for foregoing FP were the patient's attitudes, followed by the patient's clinical characteristics. For the patients, the attitude included fear of treatment delay,^28^, ^50^ a desire to focus on the treatment,^25^, ^30^, ^43^ emotional distress,^30^, ^38^, ^44^ fear of cancer recurrence,^22^, ^30^, ^38^ fear of passing on a genetic risk for cancer,^22^, ^28^, ^56^ not wanting biological children^30^, ^56^ or more children,^28^, ^30^, ^44^ and religious beliefs.^44^, ^56^ For HCPs, the attitude included the patient's emotional distress^54^ and thinking that the patient was not concerned with infertility or parenthood.^50^, ^54^

The patient's clinical characteristics (age,^22^, ^28^, ^30^, ^56^ lack of time to perform FP^25^, ^43^, ^44^, ^60^) appeared to be more critical for HCPs (27%) than patients (18%). For HCPs, the patient's clinical characteristics involved the medical condition (too ill to delay treatment) and a poor prognosis.^50^

The lack of familial and social support was in the third place for foregoing FP, for both patients (12%) and HCPs (18%). This included the absence of familial, parental, or their partner's consent for the FP, for patients^22^, ^27^, ^43^, ^55^ and HCPs.^49^, ^50^, ^55^ Patients also pointed to barriers to fertility preservation in the social environment.^56^

The high cost of the procedures was raised by both the patients (18%)^22^, ^28^, ^30^, ^38^, ^44^, ^56^ and HCPs (13%). Finally, 4% of patients forwent FP because of the clinical practice, including a lack of communication with the physician and a feeling that they had no choice.^38^, ^56^

### 3.5 Patient's perceptions, emotions, and concerns related to (potential) infertility

Concerns and psychological impacts of cancer and potential infertility were reported for prepubescent and pubescent patients and categorized into “information,” “life concerns,” “parenthood,” “health risks,” “relationships,” and “psychological impact” (Table 2).

**TABLE 2:** Patient's perceptions, emotions and concerns related to (potential) infertility. Concerns and psychological impacts of cancer and potential infertility reported by prepubescent and pubescent patients were categorized into “information”, “life concerns”, “parenthood”, “relationships”, “health risks” and “psychological impact”. The total number of articles reporting on each concern is reported, as well as the number of patients surveyed with qualitative and quantitative methods and the number of patients agreeing with such concern (available for quantitative studies only). An “X” in the column “PP + P” or “P” indicates that the studies surveyed both prepubescent (PP) and pubescent (P) patients, or only pubescent patients (P).

	Nb. of articles	Nb. of patients	Nb. of patients agreeing	PP + P	P	References
Information						
Unmet information needs	4	218	25	X		[16, 20, 44, 84]
	5	1383	28		X	[15, 16, 23, 29, 37, 38, 39]
Life concerns						
Concern about infertility risks	2	133	9	X		
	8	2245	1014		X	[19, 23, 29, 37, 66, 67, 68, 85]
Impact on life plans and future	2	76	—	X		[16, 43]
	4	1467	—		X	[3, 21, 37, 69]
Parenthood						
Desire to have children	5	2864	2517	X		[24, 43, 44, 70, 86]
	5	2774	515		X	[34, 37, 40, 69, 85]
Parenthood as part of identity as a man or woman	1	105	—	X		[17]
	3	959	—		X	[15, 21, 37]
Relationships						
Importance of childbearing/parenthood to partner	2	154	—	X		[39, 47]
	2	389	—		X	[15, 85]
Challenges in establishing relationships	5	201	—	X		[16, 18, 35, 43, 47]
	6	528	—		X	[3, 15, 39, 41, 48, 68]
Impact of cancer and fertility on social relations	1	13	—	X		[35]
	3	561	—		X	[3, 19, 69]
Health risks						
Own health risks (due to FP or pregnancy)	8	2266	—		X	[15, 19, 21, 37, 40, 41, 66, 85]
Future child's risks	4	2836	158	X		[24, 39, 44, 70]
	4	1582	215		X	[15, 37, 41, 68]
Psychological impact						
Alteration of self‐esteem, self‐confidence, identity	2	23	—	X		[35, 47]
	2	71	—		X	[3, 15]
Alteration of body image	1	13	—	X		[35]
	4	672	—		X	[3, 19, 68, 69]

Emotional distress related to infertility was frequently reported within the transcribed verbatim of patients.^13^, ^14^, ^15^, ^19^, ^35^, ^36^, ^37^, ^38^, ^39^, ^41^, ^42^, ^45^ Many patients complained of a lack of information regarding fertility.^13^, ^14^, ^18^, ^20^, ^21^, ^27^, ^35^, ^36^, ^42^ Concerns regarding infertility risks, impacts on life plans, and the future were predominant in pubescent patients.^17^, ^21^, ^27^, ^35^, ^36^, ^64^, ^65^, ^66^ As prepubescent patients were less likely to be asked, there is less documentation of their concerns in this regard.^14^, ^37^ The desire to have a child was expressed by 20% of prepubescent^32^, ^35^, ^36^, ^38^, ^67^ and 90% of pubescent patients.^15^, ^22^, ^41^, ^42^, ^68^ For some patients, parenthood can be felt as part of their identity as a man or a woman^13^, ^15^, ^19^, ^35^ and at the same time, the risk of sterility can affect the establishment of social relationships^1^, ^17^, ^33^, ^67^ and romantic relationships,^1^, ^13^, ^14^, ^16^, ^33^, ^37^, ^39^, ^41^, ^45^, ^46^, ^66^ and may disrupt the current partnership.^13^, ^36^, ^37^, ^45^

Their own health risks were reported only in patients who had cancer after puberty. Concerns about the future child's health were noted mainly in patients who had cancer after puberty and who were more afraid of transmitting the disease (215/1582, 14%).^13^, ^35^, ^39^, ^66^ Psychological health was rarely discussed, although a few studies showed that for some patients fertility impairment induced anxiety and depressive symptoms,^1^, ^13^, ^35^, ^47^ created narcissistic wounds (altered self‐esteem, self‐confidence, and identity),^1^, ^13^, ^33^, ^45^ and may alter body image.^1^, ^17^, ^33^, ^66^, ^67^

## 4 DISCUSSION

This SLR was motivated by the contrast between the low proportion of AYA preserving their fertility (banking trial for 45% boys, with a significant proportion of failures, and 23% of girls) and the large impact of fertility concerns on the quality of life of young cancer survivors. Although there are nowadays several ways to achieve parenthood, less than half of female cancer survivors^69^ and a quarter of male cancer survivors^70^ report a preference for biological children.

In the studies analyzed in this SLR, 17 articles reported on the type of FP performed and revealed a low proportion of young patients undergoing FP especially in pubescent girls and prepubescent children. In practice, for many years and in many countries, ethical and judicial reasons have limited the development of ovarian tissue cryopreservation (OTC). The patients were selected according to the very strict criteria of Edinburgh.^71^ These criteria requirements are: (a) a realistic chance of surviving for 5 years, (b) a high risk of premature ovarian insufficiency (>50%), and (c) no previous chemotherapy or radiotherapy if aged 15 years or older at diagnosis, but mild, non‐gonadotoxic chemotherapy acceptable if younger than 15 years. These criteria are currently applied in many countries, although some authors acknowledge that they warrant being updated.^72^ Moreover, worldwide, OTC could only be performed as a research protocol, especially the step of ovarian tissue auto‐transplantation in cured adult patients.^6^, ^73^

In 2020, Shapira et al.^74^ reported a pregnancy rate of 41% after ovarian tissue autotransplantation, and Dolmans et al.^75^ put the number of children born worldwide after autotransplantation at approximatively 200. Although the exact number of transplantations performed is unknown, in October 2019, this number of births convinced the experts of the American Society for Reproductive Medicine to recommend that this procedure should no longer be considered to be experimental. Other European countries have adopted a similar strategy. For instance, in France, this experimental designation was removed in June 2017 from the national guidelines of ART and fertility preservation. Two births after autotransplantation of cryopreserved prepubertal ovarian tissue have been reported.^76^, ^77^ Nevertheless, very often, when it was possible HCPs continued to include patients in research protocols before grafting because they had doubts about the safety of the procedure.

Even in countries where OTC can be carried out in current care, its performance has not undergone significant growth for two main reasons. The first is related to time constraints and the second to uncertainty surrounding the reuse of cryopreserved gametes/gametic tissues. In young cancer patients, the start of treatment is often an emergency: stimulating ovulation would require postponing the start of treatment by at least 3 weeks (2 weeks of stimulation and 1 week of post‐follicular puncture healing), which is often not possible. For prepubescent children, the reasons for the low number of FP are probably the constraints of surgical removal of gametic tissues and the uncertainty of their reuse by autotransplantation or in vitro maturation. On the one hand, in many childhood cancers, the risk of gonadal metastases is a contraindication to subsequent autotransplantation. On the other hand, in vitro maturation of infantile germ cells has not yet led to the birth of a living child, although scientific progress is continuing and promising. Time constraints and doubts about the effectiveness of the techniques influence the information provided by HCPs and the decision made by patients and their parents.

Unlike the situation with girls, referral of pubescent boys to sperm cryopreservation by health care professionals is easier and quicker and the sampling is not invasive. Utilization of frozen sperm is very common, either in artificial insemination or in in vitro fertilization, and is as effective as the use of fresh sperm and without risk for its offspring. Nevertheless, this procedure can present challenges to the young patient because it requires him to masturbate to provide a semen sample. Adolescents with a history of masturbation have higher chances of completing sperm banking.^55^ However, the high stakes of this procedure and the general psychological state of the young boy can impede his ability to masturbate. A failure of the collection at this time can result in feelings of inadequacy and low self‐esteem at a time when it is crucial for the young boy to project himself positively into the future.^78^

The care of children with cancer is constantly evolving, including fertility counseling. In their review, Logan and Anazodo^4^ acknowledge the lack of specific guidelines on how best to conduct fertility counseling with cancer patients, both at the time of cancer treatment and beyond. At each stage of care, the HCP and the fertility specialist must adapt to the psychological state of the patient and their life plan. They have to provide clear and representative information on the infertility risks and the reproductive options available. They also should be careful to avoid triggering additional stress in patients weakened by the announcement of the diagnosis of cancer and later during the remission process.

The 58 articles reviewed to understand the impact of fertility disorders revealed barriers and facilitators involved in fertility discussion and in continuing or discontinuing FP. One of the main barriers to fertility discussion was linked to the temporality: the emergency of oncological care leaves no room either for the initiation of fertility discussion or for reflection on life plans.^17^ Attitudes about parenthood change over time and patients who are further into survivorship are less burdened by concerns about cancer. Therefore, the self‐constructs that exist at the time of cancer and that are shaped by the consequences of surviving a life‐threatening illness should be considered in the global care of patients.

Infertility concerns will surface later in life in the context of relationships and family plans.^47^ Surveys of young adult cancer survivors have revealed that their expectations are similar to those of their healthy peers regarding reproductive health, expectations for romantic partnership, friendships, body image, sexuality, gender identity and orientation, fertility, contraception, and psychosexual adjustment.^79^ Therefore, infertility can be experienced as a narcissistic wound that creates a sense of emptiness.^80^ For some women, fertility is associated with the desire to appear normal.^19^ As described by Dryden et.al,^46^ some patients consider motherhood as being central to their psychological and social completeness and fulfillment. Increasing awareness and discussing fertility concerns or alternatives to biological parenthood may be beneficial to the family‐building plans of survivors'.^81^, ^82^

Nowadays, parenthood is not a life goal for everyone. However, for those who want to be a parent to a child irrespective of the biological origin of the gametes, other ways to become a parent are available: adoption, foster care, and step‐parenting. Another alternative to FP is gamete donation which can be reassuring for patients who fear transmitting their illness to their offspring. Altogether, gamete donation and adoption are viable strategies in place of or in complement to fertility preservation to enhance the quality of life of survivors who wish to have a child after cancer.^83^, ^84^ ART and/or adoption also generates distress, either by its relatively poor level of success (in case of ART), or by interaction with adoption agencies assessing the patient's potential to parent post‐cancer. In addition, financial difficulties for patients can impact the family‐building plan of young cancer survivors^85^ as ART or adoption are usually very expensive procedures and the financial aid varies according to the country.

The unknown or uncertain fertility status can lead to feelings of doubt and anxiety in some cancer survivors.^73^ Thus, for each patient, accurate assessment of the risk of infertility appears necessary, even though it is often complex because it takes into account the initial fertility, the type of cancer, and the treatment.^2^ Decisional trees are available to help in this process.^86^, ^87^ Moreover, continuous progress in Fertility Preservation procedures increases the chances of conception with ART, even with substantial gonadal damages. Therefore, updated HCPs' knowledge about FP appears indispensable as well as the inclusion of fertility specialists into the care plan, as they and the oncologists are the preferred sources of information on fertility for cancer survivors.^21^ Furthermore, the chances for prepubescent children to conceive with their own gametes increase significantly^88^, ^89^, ^90^, ^91^, ^92^, ^93^ because of: (a) the constant progression of oncology protocols taking into account reproductive toxicity, and (b) the constant optimization of the techniques of reuse of immature germ cells or germinal tissues (through autotransplantation or tissue/cells in vitro maturation). More systematic recourse to a fertility specialist could help increase access to FP for girls and prepubescent patients.

In addition to the HCP's competence, the receptiveness of the patient and their family is essential. Their state of receptivity is the consequence of their level of psychological availability and of their knowledge about fertility (initial knowledge and additional knowledge learned during hospitalization through written documents and discussions with HCPs^30^, ^31^). Family‐centered psychological interventions appear to be a better support to help patients and their families to make the best decision related to the availability of FP, according to the medical situation as well as socio‐cultural conditions/context.^94^, ^95^

However, some obstacles are economic: the practice of ovarian tissue cryopreservation is closely correlated with the country's legislation and health care funding.^73^ While in France and Canada the cost of the procedure is supported by social solidarity, most countries in the world are not as accommodating. Indeed, financial aid associations have been created, such as the Livestrong® program in the United States. However, surveys have shown that the proposed financial aid program is only partially used,^96^ which suggests that the obstacles lie elsewhere. The religious beliefs of the patient and family may also lead to refusal of FP. Jukkala^97^ and Ramstein et al.^98^ have reviewed religious considerations and point out that FP is an acceptable solution for Christianity, Islam, and Judaism for children and adolescents as it does not imply embryo storage and prevents recourse to gamete donation, which can be run counter to religious norms.^99^ It must be acknowledged that fertility preservation is not the only answer to a good quality of life, and it is not the only path to parenthood for everyone. In terms of cancerous disease, FP might not be feasible when the gonads are affected by tumor cells, or when the emergency to treat does not allow time for gametes/tissue collection. Sociocultural and ethical conditions can impede this process and should be considered in patient care, not as a reason for FP, but rather as an interpretive filter that the patient and family use to assess the information about the options available to them.^100^ In addition, masturbation for semen collection and intravaginal ultrasound monitoring of ovulation stimulation can be quite confronting, especially for prepubescent patients and their parents.

Alongside the removal of the barriers to the fertility discussion, the following facilitators could be promoted. As mentioned before, potential fertility and options for parenthood should be included alongside the oncological care for pubescent patients, both for boys and girls. Parents are also essential as a source of support from the diagnosis to the end of treatment.^33^, ^35^ Their understanding and adherence to the care plan, including fertility concerns with or without preservation measures, is essential.^45^, ^47^, ^65^, ^101^ The patient's own emotional state is also important as a negative emotional attitude tends to lead to giving up on FP. Expressing emotions related to impaired fertility and an uncertain future helps with coping and reduces distress.^102^ In addition, formulation of a parenting plan suggests a promise of healing, whereas the absence of FP as an option could be interpreted by patients and their families as a logical consequence of a poor short‐term prognosis. Thus, the intervention of a psychologist should focus on the child's best interest^103^ and participate in decreasing the patients' and the families' distress, and increase their receptiveness to fertility discussion with an oncologist or an FP specialist. Moreover, in case of refusal of FP, it may help to decipher whether this refusal is absolute and related to a real lack of desire for a child or whether it is an afterthought that may lead to patients regretting their decision later in life.^9^, ^44^

## 5 METHODOLOGICAL LIMITATIONS

First, in our methodology, we chose to accept the heterogeneity of the articles analyzed to better define and understand the mechanisms of the pathways that lead to FP as a whole, and it was, therefore, not possible to avoid methodological biases and lack of consensus.

Considering only the quantitative data, the questionnaires used are mostly not standardized because there are no specific questionnaires to assess the impact of fertility on the quality of life of young cancer survivors, except for the Reproductive Concerns After Cancer scale for adult patients.^104^ Therefore, a few teams have created their own questionnaires or relied on interviews and focus groups where other biases can be expected, such as interpretation bias and desirability bias. Secondly, in studies in which patients and parents return a questionnaire, it is impossible to know who, i.e., the patient or the parents, actually answered the questionnaires, which may be an important bias in exploring the concerns expressed by the patients.

Thirdly, in this SLR, the term HCPs refers to health care professionals because of the diversity of the surveyed population representing physicians (oncologists, pediatricians, fertility specialists, endocrinologists, etc.), nurses, and social workers. When analyzing their opinions, a bias was found in the description of their practice when it was not the actual numbers provided but their approximate opinion. Another difficulty arose when a professional speaks for an entire institution,^40^, ^53^, ^54^ which may not fully represent the whole clinical practice.

Finally, the age of the patients was never consensual among the studies (Table S2), and it was not possible to differentiate the patients based on their age or pubertal status (rarely defined). The editors of JAYA oncology recommend subdividing the AYA population into 15–18 (early young adulthood), 19–24 (young adulthood), and 25–39 (late young adulthood) years of age,^105^ implying that the physiological and psychosocial maturity and life objectives are oriented differently. In this SLR, a 25‐year cut‐off was chosen for consistency in patient psychology. After 25 years, patients are more frequently engaged in romantic relationships, which generally greatly modifies their concerns.^81^ Fertility is a more concrete concept (they may have already either used contraception, experienced an unwanted pregnancy, or have already desired to have a child). Studies that also included older patients were retained to adhere to the ‘systematic’ nature of the review focused on childhood and adolescent cancer/AYA survivors.

## 6 CONCLUSIONS AND FUTURE DIRECTIONS

This SLR is an update on the reality of fertility preservation issues among patients and professionals. Although recommendations worldwide^4^ call for fertility to be presented during or after treatment, the reality is different, especially since HCPs either do not have guidelines^52^, ^61^ or are reluctant to seek guidelines^49^, ^59^ that are not always adapted to very young patients. Moreover, guidelines specific to prepubescent patients are lacking.

**FIGURE 4:**
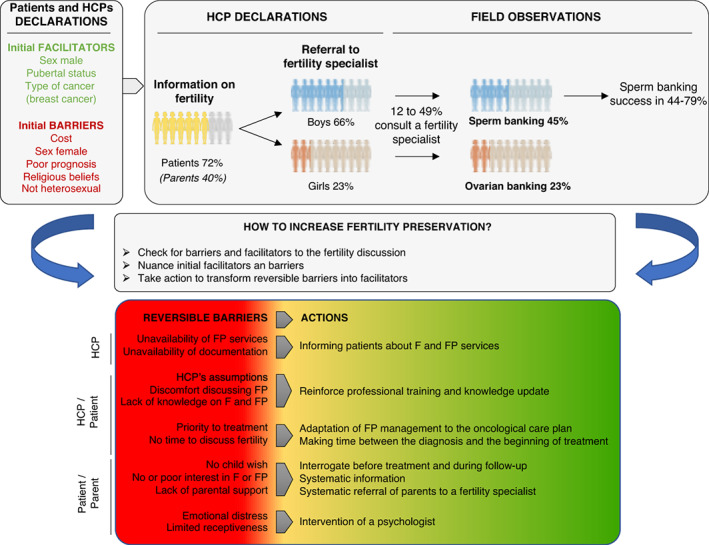
Summary and concrete propositions to increase fertility preservation. The top panel reports the proportion of patients/parents informed/referred to a fertility specialist/ultimately carrying out gamete/tissue banking. The middle panel offers a systematic analysis of the elements at play in patient/parent care and status, leading to a proposal for action (bottom panel) affecting the HCPs, the HCP/patient relationship, and the patients themselves.

In response to the questions presented in the introduction, this SLR showed that patients' and professionals' knowledge of FP is heterogeneous and that banking is low (Figure 4). Fertility discussion is facilitated when patients are pubescents, or male or have breast cancer (for older AYA). Although removable, exclusive barriers to FP were found to be the cost, female gender, prognosis, religious beliefs, and sexual orientation, none of these should refrain HCPs from discussing FP. Improving banking requires removing the reversible barriers, starting by thoroughly informing parents and patients throughout their care. To facilitate the decision‐making process, Wang et al. reviewed existing patient decision support tools.^106^ They showed that they improved fertility knowledge and reduced decisional conflict. However, although Allingham et al. have proposed a decision aid for parents of children and adolescents with cancer,^107^ none exists specifically for children to date.

By reinforcing professional training, HCPs should feel more comfortable discussing FP and more inclined to provide adequate information to the families. Understandably, treatment is a priority, however the time constraint should not be a barrier to discuss fertility and other alternatives to having children. Systematic information and systematic referral to a fertility specialist alongside oncological care would ensure that parents and patients are well‐informed.

Achieving FP is an uphill battle and requires multi‐disciplinary teams working with the patients and families from the diagnosis of cancer to long‐term follow‐up. We suggest that (a) each medical team is partnered with a fertility specialist, (b) the medical team is educated about fertility issues, (c) practical training is offered to help HCPs become more comfortable discussing fertility, (d) each patient treated for cancer is provided with age‐appropriate materials, and (e) psychological counseling addressing fertility issues is offered.

## AUTHOR CONTRIBUTIONS

**Karima El Alaoui‐Lasmaili:** Conceptualization (equal); data curation (lead); investigation (lead); methodology (lead); project administration (equal); visualization (equal); writing – original draft (lead); writing – review and editing (equal). **Phi linh Nguyen‐Thi:** Formal analysis (supporting); methodology (equal). **Nadine Demogeot:** Resources (equal); supervision (equal); validation (equal). **Joëlle Lighezzolo‐Alnot:** Resources (equal); supervision (equal); validation (equal). **Marie José Gross:** Resources (equal); validation (equal). **Ludovic Mansuy:** Resources (equal); validation (equal). **Pascal Chastagner:** Funding acquisition (lead); resources (equal); validation (equal). **Isabelle Koscinski:** Conceptualization (lead); formal analysis (equal); investigation (equal); project administration (equal); supervision (lead); validation (equal); visualization (equal); writing – original draft (equal); writing – review and editing (lead).

## FUNDING INFORMATION

Institut National du Cancer (INCa, France).

## CONFLICTS OF INTEREST

The authors have no conflicts of interest to declare.

## ETHICS APPROVAL STATEMENT

Ethics approval was not required for this systematic literature review.

## INFORMED CONSENT

Informed consent was not required for this systematic literature review.

## Supporting information


Table S1

Table S2

Table S3

Table S4

Table S5

## Data Availability

N/A
