# Supplementary material for: Fertility discussions and concerns in childhood cancer survivors, a systematic review for updated practice
Source: Cancer Med. 2022 Oct 12;12(5):6023–39. doi: 10.1002/cam4.5339 (PMC10028046; doi:10.1002/cam4.5339)
Supplement: Supplementary file 1 — Table S1 [file CAM4-12-6023-s002.docx]

Supplementary table 1: Age at diagnosis and age at research reported in the inclusion criteria of the articles included in this SLR.

| Ref | **Author** | **Country** | **Date** | **Type of study** | **Patient/Parent/HCP** | **Method** | Patient | Nb males | Nb females | Nb Parent | Nb HCP | Age at research | SD | Age at diagnosis | SD |
| --- | --- | --- | --- | --- | --- | --- | --- | --- | --- | --- | --- | --- | --- | --- | --- |
| ^61^ | Panagiotopoulou | United Kingdom | 2017 | Quantitative | HCP | survey |  |  |  |  | 48 | - | - | <18 | - |
| ^28^ | Shnorhavorian | USA | 2015 | Mixed-Methods | Parent | survey | 459 | 283 | 176 | - | - | - | - | 15-39 | - |
| ^35^ | Ussher | Australia | 2018 | Mixed-Methods | Patient | survey and interview | 878 | 185 | 693 | - | - | 42,5 | 14,2 | not targeted | - |
| ^57^ | Rosenberg | USA | 2017 | Quantitative | HCP | survey | - |  |  | - | 93 | <42 |  |  |  |
| ^49^ | Vadaparampil | USA | 2008 | Qualitative | HCP | interview | - |  |  | - | 24 | - | - | 0 - 21 | - |
| ^58^ | Vadaparampil | USA | 2007 | Quantitative | HCP | survey | - |  |  | - | 115 | - | - | - | - |
| ^53^ | Yee | Canada | 2012 | Quantitative | HCP | survey | - |  |  | - | 25 | - | - | - | - |
| ^50^ | Kohler | USA | 2011 | Quantitative | HCP | survey | - |  |  | - | 209 | - | - | - | - |
| ^56^ | Klosky | USA, Canada | 2018 | Quantitative | Patient | survey | 146 | 146 |  | - | - | 16,5 | 2 | 13-21 | - |
| ^48^ | Armuand | Sweden | 2012 | Quantitative | Patient | survey | 484 | 156 | 328 | - | - | 41,2 (21-53) | - | 36,2  (18-45) | - |
| ^34^ | Bastings | The Netherlands | 2014 | Quantitative | Patient and HCP | survey | 1169 |  | 1169 |  | 103 | 27,1 | 7,3 | 0-39 | - |
| ^51^ | Garrido-Colino | Spain | 2017 | Quantitative | HCP | survey | - |  |  | - | 50 | - | - | - |  |

Supplementary table 1 continued: Age at diagnosis and age at research reported in the inclusion criteria of the articles included in this SLR.

| ^60^ | Kapadia | USA | 2020 | Quantitative | Patient | survey | 82 | 82 | 82 | - | - | - |  | 16  (13-25) |  |
| --- | --- | --- | --- | --- | --- | --- | --- | --- | --- | --- | --- | --- | --- | --- | --- |
| ^52^ | Terenziani | Europe | 2014 | Quantitative | HCP | survey | - |  |  | - | 64 | - |  |  |  |
| ^54^ | Diesch | Switzerland | 2016 | Quantitative | HCP | survey |  |  |  |  | 9 | - |  | < 18 |  |
| ^63^ | Ju | China | 2019 | Quantitative | HCP | survey |  |  |  |  | 184 | - |  |  |  |
| ^31^ | Klosky | USA, Canada | 2017 | Quantitative | Patient | survey | 99 | 99 |  | - | 52 |  |  | 13-21 |  |
| ^62^ | Klosky | USA, Canada | 2017 | Quantitative | Patient and parent | survey | 146 | 146 |  | 144 |  |  |  | 13-21 |  |
| ^55^ | Klosky | USA, Canada | 2017 | Quantitative | Patient, Parent and HCP | survey | 146 | 146 |  | 144 | 52 |  |  | 13-21 |  |
| ^59^ | Adams | United Kingdom | 2013 | Quantitative | HCP | survey | - |  |  | - | 100 |  |  |  |  |
| ^29^ | Skaczkowski | Australia | 2018 | Quantitative | Patient | survey | 207 | 87 | 120 | - | - | 21,0 | 3,0 | 15-24 |  |
| ^40^ | Chong | Canada | 2010 | Quantitative | HCP | survey | - |  |  | - | 15 | - | - |  |  |
| ^22^ | Hohmann | Germany | 2011 | Quantitative | Patient | survey | 2489 | 1167 | 1322 | - | - | 25,7  (19-43) | 5,3 | 8,1 | 4,3 |

Supplementary table 1 continued: Age at diagnosis and age at research reported in the inclusion criteria of the articles included in this SLR.

| ^23^ | Benedict | USA | 2016 | Quantitative | Patient | interview and focus group | 346 |  | 346 |  |  | 29,9 (18-35) | 4,1 | 23,6 (0-35) | 7,5 |
| --- | --- | --- | --- | --- | --- | --- | --- | --- | --- | --- | --- | --- | --- | --- | --- |
| ^108^ | Nahata | USA | 2018 | Quantitative | Patient | survey | 45 | 45 |  | - | - | 19,5 (15-25) | 2,6 | 14,2 | 3,6 |
| ^25^ | Wyns | Belgium | 2015 | Quantitative | Patient and parent | survey | 120 | 120 |  | 120 | - | 12-18  <12 |  | 14,4 6,05 | 1,5 3,7 |
| ^16^ | Lehmann | USA | 2018 | Quantitative | Patient | survey | 92 | 34 | 58 | - |  | 22-44 |  | (5-18) |  |
| ^33^ | Kieffer | France | 2012 | Qualitative | Patient | interview | 13 | 5 | 8 | - |  | 23,7  (16-29) |  | (9-14) |  |
| ^109^ | Kim | USA | 2015 | Quantitative | Patient | survey | 56 |  | 56 | - |  | 26,0 | 4,0 | 13,0 | 6,0 |
| ^26^ | Ferrante | USA | 2019 | Quantitative | Patient | survey | 45 | 45 |  | - |  | 18,98 (15-25) | 2,6 | 14,2 | 3,6 |
| ^14^ | Ellis | Australia | 2016 | Qualitative | Patient and parent | interview | 19 | 13 | 6 | 97 |  | 16,1  (12-20) | 2,2 | 13,3 | 2,6 |
| ^42^ | Sandheinrich | USA | 2018 | Quantitative | Patient and parent | survey | 26 |  | 26 | 23 |  | 16  (13-18) |  | 0-18 |  |
| ^18^ | Van den Berg | The Netherlands | 2008 | Quantitative | Parent | survey | 117 |  |  | 202 |  | 13  (3-24) |  | 6,4  (0-17) |  |

Supplementary table 1 continued: Age at diagnosis and age at research reported in the inclusion criteria of the articles included in this SLR.

| ^27^ | Patel | USA | 2019 | Quantitative | Patient | survey | 47 | 28 | 19 | - | - | 15  (12-20) | 2,1 | ≥12 |  |
| --- | --- | --- | --- | --- | --- | --- | --- | --- | --- | --- | --- | --- | --- | --- | --- |
| ^30^ | Vogt | Australia | 2018 | Mixed-Methods | Patient | survey and interview | 23 |  | 23 | - | - | 29,2 (16-39) | 6,3 | 16-40 |  |
| ^43^ | Baysal | The Netherlands | 2015 | Mixed-Methods | Patient | survey and interview | 87 |  | 87 | - | - | 28,3 | 5,9 | 0-42 |  |
| ^44^ | Benedict | USA | 2015 | Quantitative | Patient | survey | 159 |  | 159 | - | - | 34,8 (22-43) | 5,1 | 32,8 (20-39) | 5 |
| ^21^ | Johnson | USA | 2018 | Quantitative | Patient | survey | 69 | 30 | 39 | - |  | 19,6  (12-25) | 3,9 | ≤18 |  |
| ^13^ | Benedict | USA | 2016 | Qualitative | Patient | interview and focus group | 43 | 18 | 25 | - |  | 19,6 | 2,8 | 15,4  (14-21) | 1,5 |
| ^19^ | Crawshaw | USA | 2010 | Qualitative | Patient | interview | 38 | 17 | 21 | - |  | 21  (16-30) |  | 15 (13-30) |  |
| ^64^ | Young | USA | 2019 | Quantitative | Patient | survey | 747 |  | 747 | - |  | 33,1 (18-40) | 5,1 | 15-35 |  |
| ^32^ | Geue | Germany | 2014 | Quantitative | Patient | survey | 149 | 50 | 99 | - |  | M 32,1 W 34,6 (18-45) | 7,5 6,8 | >18 |  |
| ^66^ | Ljungman | Sweden | 2019 | Quantitative | Patient | survey | 111 | 111 |  | - |  | 32,1 | 5,5 | 30,1  (16-39) | 5,6 |

Supplementary table 1 continued: Age at diagnosis and age at research reported in the inclusion criteria of the articles included in this SLR.

| ^17^ | Nieman | USA | 2007 | Qualitative | Patient and parent | focus group | 10 |  | 10 | 10 |  | 32,6  (23-36) |  | 14,5  (13-21) |  |
| --- | --- | --- | --- | --- | --- | --- | --- | --- | --- | --- | --- | --- | --- | --- | --- |
| ^41^ | Lehmann | USA | 2019 | Qualitative | Patient | interview | 57 | 19 | 38 |  |  | 29,8 (20-40) | 4,4 | 11,8 | 3,7 |
| ^15^ | Lehmann | USA | 2017 | Quantitative | Patient | survey | 105 | 52 | 53 | - |  | 26,5 (20-40) | 4,9 | 11,5  (5-18) | 3,7 |
| ^45^ | Stinson | Canada | 2015 | Qualitative | Patient and parent | interview | 20 | 9 | 11 | 20 |  | 15  (12-17) | 1,8 | 13,2  (8-16) | 2,5 |
| ^47^ | Stein | USA | 2014 | Qualitative | Patient and parent | focus group | 15 | 15 |  | 7 |  | 35  (18-55) |  | 14  (10-21) |  |
| ^1^ | Lehmann | Sweden | 2014 | Qualitative | Patient | interview | 28 | 15 | 13 |  |  | 25,5  (23-29) | 1,5 | 13-19 |  |
| ^67^ | Bellizzi | USA | 2012 | Quantitative | Patient | survey | 523 | 194 | 329 |  |  |  |  | 15-39 |  |
| ^38^ | Parton | Australia | 2019 | Mixed-Methods | Patient | survey and interview | 878 | 185 | 693 | - | - | 42,5 | 14,2 |  |  |
| ^46^ | Dryden | Australia | 2014 | Mixed-Methods | Patient | survey and interview | 8 | 8 |  |  |  | 23,1 | 2,4 | 18-26 |  |

Supplementary table 1 continued: Age at diagnosis and age at research reported in the inclusion criteria of the articles included in this SLR.

| ^39^ | Gorman | USA | 2015 | Quantitative | Patient | survey | 204 | 204 |  |  |  | 28,4 (18-35) | 4,4 |  |  |
| --- | --- | --- | --- | --- | --- | --- | --- | --- | --- | --- | --- | --- | --- | --- | --- |
| ^102^ | Razzano | Italy | 2014 | Mixed-Methods | Patient | survey | 48 |  | 48 |  |  | 28,0 | 7,8 | 18-40 |  |
| ^36^ | Benedict  Support care | USA | 2018 | Quantitative | Patient | survey and interview | 346 |  | 346 |  |  | 29,9 (18-35) | 4,1 |  |  |
| ^65^ | Oosterhuis | USA | 2008 | Quantitative | Patient and parent | survey | 37 | 20 | 17 | 97 |  |  |  |  |  |
| ^68^ | Raghunathan | USA | 2018 | Quantitative | Patient | survey | 187 |  | 187 |  |  | 29,5 (18-35) | 4,1 | 23,7 (0-34) | 7,2 |
| ^37^ | Nilsson | Sweden | 2014 | Qualitative | Patient | focus group | 134 | 66 | 68 |  |  | 21  (16-24) |  | 8  (0-17) |  |
