# Supplementary material for: Fertility discussions and concerns in childhood cancer survivors, a systematic review for updated practice
Source: Cancer Med. 2022 Oct 12;12(5):6023–39. doi: 10.1002/cam4.5339 (PMC10028046; doi:10.1002/cam4.5339)
Supplement: Supplementary file 2 — Table S2 [file CAM4-12-6023-s001.docx]

Supplementary table 2: Experience of fertility issues. Patients’ and parents’ knowledge and experience of fertility is reported, as well as the technique of fertility preservation that was pursued. The total number of articles reporting on each criterion is presented, as well as the number of patients and parents surveyed with qualitative and quantitative methods. The columns entitled “agreeing” inform on the number of patients and parents who indicated agreeing with each criterion in the surveys (available for quantitative studies only).

|  | Nb of articles | Nb of eligible patients | Patient agreeing | Nb of eligible parents | Parents agreeing | Ref. |
| --- | --- | --- | --- | --- | --- | --- |
| Had knowledge about their own or child’s reproductive health | 9 | 549 | 94 | 309 | 5 | ^13–19,21,108^ |
| Discussed potential infertility | 17 | 4419 | 1814 | 322 | - | ^13,16,18,21–23,25–33,108,109^ |
| Discussed FP options before treatment | 10 | 2218 | 1079 | 23 | - | ^17,27–30,42,44,48,60,64^ |
| Were referred to a FP specialist | 9 | 2139 | 491 | - | - | ^15,29–32,34,40,44,108^ |
| Underwent FP | 17 | 3051 | 645 | - | - | ^13,18,19,23,25,27,28,30,32,40,43,44,48,60,64,66,109^ |
| Sperm banking | 9 | 731 | 329 | - |  | ^13,19,26,27,31,32,40,48,56^ |
| Oocyte preservation | 6 | 1797 | 225 | - | - | ^27,30,32,34,44,48^ |
| Ovarian tissue cryopreservation | 2 | 1267 | 145 | - | - | ^32,34^ |
| Embryo preservation | 3 | 1521 | 505 | - | - | ^30,34,48^ |
| Shielding/Transposition | 1 | 1169 | 164 | - | - | ^34^ |
| Gonadotropin hormone, ovarian suppression | 4 | 605 | 24 | - | - | ^27,32,44,48^ |
| Underwent fertility testing | 2 | 137 | 62 | - | - | ^16,26^ |
