# Supplementary material for: Fertility discussions and concerns in childhood cancer survivors, a systematic review for updated practice
Source: Cancer Med. 2022 Oct 12;12(5):6023–39. doi: 10.1002/cam4.5339 (PMC10028046; doi:10.1002/cam4.5339)
Supplement: Supplementary file 3 — Table S3 [file CAM4-12-6023-s005.docx]

Supplementary Table 3: HCP recommendations for practice and retrospective point of view

| Ref. | ^49^ | ^50^ | ^51^ | ^52^ | ^53^ | ^54^ | ^31^ | ^55^ | ^40^ |
| --- | --- | --- | --- | --- | --- | --- | --- | --- | --- |
| Type | Declarative practice | Declarative practice | Declarative practice | Declarative practice | Declarative practice | factual | factual | factual | factual |
| Nb. of HCP | 24 | 209 | 50 | 64 | 25 | 9 | 52 | 52 | 15 |
| Discussed potential infertility | - | - | - | - | - | - | 71,70% | - | 40% with parents |
| Discussed FP options before treatment | Not for PP | Yes | - | - | - | - | - | - |  |
| Written information | - | - | - | - | 44% | - | - | - | 60% |
| Referred to FP specialist | - | 66% M 23% W | 12% if interested 38% only P | - | - | 12% | 18,20%  (18/99) | - | 49% (85/172 males in 2 years) |
| underwent FP | - | - | - | - | - | 12% | - | - | 38/85 |
| Sperm banking | - | 85% | 96% P | 59% for all  41% selected cases | Yes | 88% | 40/54  ( (59,3% success) | 53,4% (43,8% success) | success in 30/38 =17% (on total of 172) |
| Oocyte preservation | - | - | 4% PP 40% P | 33% | Yes | - | - | - | - |
| Testicular tissue cryopreservation | not discussed | 10% | 4% PP 36% P | 12,5% in selected cases | - | - | - | - | - |
| Ovarian tissue cryopreservation | not discussed | 13% | 36% for PP girls | 62,5% suggest | Yes | 47% | - | - | - |
| Embryo preservation | - | - | - | 33% | Yes | - | - | - | - |
| Shielding/Transposition | - | - | - | 37% Testicular  79,5% ovarian T | - | - | - | - | - |
| Gonadotropin hormon, ovarian suppression | - | - | - | 48% | - | 42% | - | - | - |
