# Supplementary material for: Fertility discussions and concerns in childhood cancer survivors, a systematic review for updated practice
Source: Cancer Med. 2022 Oct 12;12(5):6023–39. doi: 10.1002/cam4.5339 (PMC10028046; doi:10.1002/cam4.5339)
Supplement: Supplementary file 4 — Table S4 [file CAM4-12-6023-s004.docx]

Supplementary Table 4: Description of the facilitators and barriers to the fertility discussion. The column ‘comments” explains why the item is considered a facilitator (F) or a barrier (B). abbreviation: HCP : Health Care Professional; P: pubescents, PP: prepubescents

|  | Facilitator (F) of Barrier (B) | Nb. of  patients | Boys | Girls | No. of HCP | P + PP | P only | Comments | Ref. |
| --- | --- | --- | --- | --- | --- | --- | --- | --- | --- |
| Age | F | -  -  484  1169 | -  -  156  - | -  -  328  1169 | 209  15  103 | X  X | X  X | Threshold to discuss 12.6 years  Threshold to discuss > 12 years  Threshold to discuss < 35 years  Threshold to discuss 20-29 years | ^50^  ^40^  ^48^  ^34^ |
|  | B | 459  878  1169 | 283  185  - | 176  693  1169 | 48  103 | X | X  X  X | -  Young age  Assumptions on age  < 20 years | ^61^  ^28^  ^35^  ^34^ |
| Prepubescent | F |  |  |  | 24 | X |  | Infertility discussed, not FP | ^49^ |
|  | B | 146 | 146 | - | - |  | X | Low Tanner stage | ^56^ |
| Pubescent | F | 146 | 146 | - | 24  226  52 | X  X | X | FP discussed with pubescents  High Tanner stage  High Tanner stage | ^49^  ^50^ ^40^  ^5655^ |
|  | B |  |  |  |  |  |  |  |  |
| Sex Male | F | -  484  207 | -  156  87 | -  328  120 | 24  -  - | X | X  X | 100% discussion  Discussion 14x more likely than female  - | ^49^  ^48^  ^29^ |
|  | B |  |  |  |  |  |  |  |  |
| Sex Female | F |  |  |  |  |  |  |  |  |
|  | B | -  484  207 | -  156  87 | -  328  120 | 24  -  - | X | X  X | Unfamiliarity with FP options  Discussion 14x less likely than male  - | ^49^  ^48^  ^29^ |
| Relationship status | F | - | - | - | 115 | X |  | If patient engaged or married | ^58^ |
|  | B | - | - | - | 100 |  | X | Patient single | ^59^ |

Supplementary Table 4 continued: Description of the facilitators and barriers to the fertility discussion.

| Sexual orientation | F |  |  |  |  |  |  |  |  |
| --- | --- | --- | --- | --- | --- | --- | --- | --- | --- |
|  | B | -  - | -  - | -  - | 115  100 | X | X | Less likely if homosexual  Less likely if homosexual | ^58^  ^59^ |
| Type of cancer | F | -  -  484  1169  82  207 | -  -  156  -  82  87 | -  -  328  1169  -  120 | 15  93  -  103  -  - | X | X  X  X  X  X | Lymphoma  Breast cancer  Leukemia  Breast cancer, Lymphoma  Hodgkin lymphoma, Leukemia  Hemato, Sarcoma, Germ cell | ^40^  ^57^  ^48^  ^34^  ^60^  ^29^ |
|  | B | 459  1169  - | 283  -  - | 176  1169  - | -  103  100 |  | X  X  X | Lymphoma, ALL, Sarcoma  Neuro, H&N, lung, urinary, thyroid, adrenal gland, eye  Hormonally sensitive cancer | ^28^  ^34^  ^59^ |
| General state of health | F | -  207  - | -  87  - | -  120  - | 50  -  15 | X  X | X | 34% patient referral  Treatment with CT and or RT  Pelvic RT, Bone marrow, CT | ^51^  ^29^  ^40^ |
|  | B | -  -  -  82  207 | -  -  -  82  87 | -  -  -  -  120 | 48  24  216  100  - | X  X | X  X  X | Medical condition  Infertility discussed, not FP  Needing immediate treatment  Needing immediate treatment  Treatment with surgery alone | ^61^  ^49^  ^50^ ^40^  ^6059^  ^29^ |
| Poor prognosis | F |  |  |  |  |  |  |  |  |
|  | B | -  -  -  - | -  -  -  - | -  -  -  - | 93  115  209  100 | X  X | X  X | 16% never discuss, 58% no FP  Less likely to discuss  Barrier to sperm banking  88% influenced by prognosis | ^57^  ^58^  ^50^  ^59^ |
| HCP’s knowledge of F and FP options, efficacy | F | -  - | -  - | -  - | 100  50 | X | X | 64% knowledge on sperm cryopreservation  78% knowledge of treatments impacts on fertility | ^59^  ^51^ |
|  | B | -  -  82  - | -  -  82  - | -  -  -  - | 48  259  184  100 | X  X | X  X | 77% knowledge gaps  Lack of knowledge of FP  Lack of knowledge of FP  Poor knowledge on tissue cryopreservation | ^61^  ^5051^  ^6063^  ^59^ |

Supplementary Table 4 continued: Description of the facilitators and barriers to the fertility discussion.

| Guidelines | F | - | - | - | 93 |  | X | 93% follow recommendations | ^57^ |
| --- | --- | --- | --- | --- | --- | --- | --- | --- | --- |
|  | B | -  -  -  -  - | -  -  -  -  - | -  -  -  -  - | 48  24  64  100  15 | X  X  X  X | X | No trust protocols in 67%  Not aware of FP guidelines  No specific guidelines in 53%  No guidelines consultation in 23%  No adolescent focused guidelines | ^61^  ^49^  ^52^  ^59^  ^40^ |
| Written info for patients | F | -  -  - | -  -  - | -  -  - | 93  25  100 |  | X  X  X | 74% provide resources on fertility  44% use published brochures  38% provide written information | ^57^  ^53^  ^59^ |
|  | B | -  -  -  -  - | -  -  -  -  - | -  -  -  -  - | 48  24  115  25  184 | X  X  X | X  X | No adequate information in 66%  Need more educational material  Need more educational material  No adequate information in 28%  Absence of specialist in 50% | ^61^  ^49^  ^58^  ^53^  ^63^ |
| Comfortability discussing FP | F | -  99  - | -  99  - | -  -  - | 209  52  100 | X | X  X | Overlook own discomfort  Comfortability with patients and parents  Comfortability in 65% | ^50^  ^31^  ^59^ |
|  | B | -  878  -  - | -  185  -  - | -  693  -  - | 48  -  24  15 | X  X  X | X | Discomfort in 39%  Discomfort  Comfortable but not trained  Lack of training | ^61^  ^35^  ^49^  ^40^ |
| Time constraint | F | - | - | - | 93 |  | X | Time constraint not an issue | ^57^ |
|  | B | -  -  -  - | -  -  -  - | -  -  -  - | 48  209  9  15 | X  X  X  X |  | Barrier in 41% HCP and 74% patients  Lack of time for female patients  Lack of time  - | ^61^  ^50^  ^54^  ^40^ |

Supplementary Table 4 continued: Description of the facilitators and barriers to the fertility discussion.

| Availability of FP services | F | -  -  245  - | -  -  245  - | -  -  -  - | 93  115  52  100 | X | X  X  X | Referral to specialist in 97%  Not a problem for 27%  Referral to FP specialist  Referral to FP specialist in 67% | ^57^  ^58^  ^3155^  ^59^ |
| --- | --- | --- | --- | --- | --- | --- | --- | --- | --- |
|  | B | -  -  -  -  1169  -  -  -  - | -  -  -  -  -  -  -  -  - | -  -  -  -  1169  -  -  -  - | 48  24  115  25  103  50  184  100  15 | X  X  X  X  X | X  X  X  X | No FP services in 35%  No link to FP specialists in 50%  Difficulty finding facilities in 37%  Poor or late referral to FP  Poor knowledge of FP facilities  No reproductive unit in 13%  FP services in 20%  No knowledge of FP facilities in 18%  Poor access to FP facilities | ^61^  ^49^  ^58^  ^53^  ^34^  ^51^  ^63^  ^59^  ^40^ |
| Emotions | F | -  - | -  - | -  - | 24  9 | X  X |  | FP as a sign of hope  Relief and coping | ^49^  ^54^ |
|  | B | -  146  -  - | -  146  -  - | -  -  -  - | 24  -  9  15 | X  X  X | X | Embarrassment for men, distraught for women  Fear of passing down cancer  Distress to life threat  Too stressed to discuss banking | ^49^  ^56^  ^54^  ^40^ |
| Desire for children | F | -  484  - | -  156  - | -  328  - | 115  -  100 | X | X  X | -  -  Importance of desire for children for HCPs | ^58^  ^48^  ^59^ |
|  | B | -  -  146 | -  -  146 | -  -  - | 24  209  - | X  X | X | Male patients less interested  Not concerned with parenthood  No desire for biological children in 5.9% | ^49^  ^50^  ^56^ |

Supplementary Table 4 continued: Description of the facilitators and barriers to the fertility discussion.

| Patient’s and family’s interest for F and/or FP | F | -  -  146 | -  -  146 | -  -  - | 24  9  52 | X  X | X | Female patients more interested  Patient and parent’s interest  Adolescent’s perception of banking benefits | ^49^  ^54^  ^55^ |
| --- | --- | --- | --- | --- | --- | --- | --- | --- | --- |
|  | B | -  -  146  1169  - | -  -  146  -  - | -  -  -  1169  - | 48  93  -  -  9 | X  X | X  X  X | Lack of interest in 82% of patients  Not wanting to discuss FP in 27%  Lack of communication from physician in 8%  Patient not asking  Lack of interest | ^61^  ^57^  ^56^  ^34^  ^54^ |
| Patient’s and family’s receptiveness | F | - | - | - | 24 | X |  | Encouraging parents to consider FP | ^49^ |
|  | B | -  -  -  146  -  - | -  -  -  146  -  - | -  -  -  -  -  - | 24  115209  -  9  15 | X  X  X  X  X | X | Delaying the discussion with parents in shock  < 50% families interested  Not concerned with fertility  Banking unnecessary for 29.5% patients  Parents overwhelmed  Adolescents not comfortable | ^49^  ^58^  ^50^  ^56^  ^54^  ^40^ |
| HCP's assumptions | F | -  - | -  - | -  - | 24  209 | X  X |  | 50% believe parents want to discuss  80% HCPs concerned about fertility risks | ^49^  ^50^ |
|  | B | 878  -  -  -  146  146  -  - | 185  -  -  -  146  146  -  - | 693  -  -  -  -  -  -  - | -  93  24  115  -  52  100  15 | X  X  X | X  X  X  X  X | HCP’s inaccurate assumptions  Risks of FP outweighed benefits  Belief that parents are uncomfortable discussing FP  Belief that FP may upset parents  Poor patient knowledge of FP  Poor patient knowledge of FP  Own perception of FP’s unsuccess  Belief adolescents too young | ^35^  ^57^  ^49^  ^58^  ^56^  ^55^  ^59^  ^40^ |

Supplementary Table 4 continued: Description of the facilitators and barriers to the fertility discussion.

| Priority to treating VS discussing FP | F | -  -  - | -  -  - | -  -  - | 93  24  100 | X | X  X | High priority FP discussion in 88%  High priority FP discussion in ¼  High priority FP discussion in 59% | ^57^  ^49^  ^59^ |
| --- | --- | --- | --- | --- | --- | --- | --- | --- | --- |
|  | B | -  -  -  - | -  -  -  - | -  -  -  - | 93  24  25  100 | X | X  X  X | Priority to cancer over FP in 77%  Priority to cancer over FP in 50%  Priority to cancer over FP in 100%  Priority to cancer over FP in 67% | ^57^  ^49^  ^53^  ^59^ |
| Religious/Moral | F |  |  |  |  |  |  |  |  |
|  | B | -  146 | -  146 | -  - | 24  - | X | X | Issue regarding masturbation in 1/3  Barrier in 5.9% | ^49^  ^56^ |
| Parent's consent / encouragement | F | 146 | 146 | - | 52 |  | X | Parents recommendation to bank | ^55,56,62^ |
|  | B | 878  -  -  -  - | 185  -  -  -  - | 693  -  -  -  - | -  24  209  64  15 | X  X  X  X | X | Discussion directed to parents  Discussion directed to parents  Absence of parental consent  Need additional permissions  Absence of parental consent | ^35^  ^49^  ^50^  ^52^  ^40^ |
| Cost | F |  |  |  |  |  |  |  |  |
|  | B | 459  -  -  -  146  -  - | 283  -  -  -  146  -  - | 176  -  -  -  -  -  - | -  93  24  209  -  100  15 | X  X  X | X  X  X  X | No insurance  Issue in 47% of patients  Cost for FP and storage  -  Issue in 5.9%  Issue in 27%  - | ^28^  ^57^  ^49^  ^50^  ^56^  ^59^  ^40^ |
