# Supplementary material for: Fertility discussions and concerns in childhood cancer survivors, a systematic review for updated practice
Source: Cancer Med. 2022 Oct 12;12(5):6023–39. doi: 10.1002/cam4.5339 (PMC10028046; doi:10.1002/cam4.5339)
Supplement: Supplementary file 5 — Table S5 [file CAM4-12-6023-s003.docx]

Supplementary Table 5: The reasons for pursuing or foregoing fertility preservation (FP) were sorted into 6 categories and presented as the point of view of patients and health care professionals (HCP).

| REASONS FOR PURSUING FP | | PP | PP + P | No. of HCP | No. of patients | Ref. |
| --- | --- | --- | --- | --- | --- | --- |
| Clinical practice | Encouraging parents | X |  | 24 | - | ^49^ |
|  | Encouraging the patient to participate in FP consultation |  | X | 52 | 266 | ^25,55^ |
|  | Consultation with fertility specialist |  | X | 155 | 415 | ^30,31,43,44,55,56^ |
| Familial & Social Support | Parent recommendation |  | X | 52 | 266 | ^25,55,56^ |
|  | Support and resources |  | X | - | 87 | ^43^ |
| Patient's clinical characteristics | Higher Tanner stage (≥3) |  | X | 52 | 146 | ^55,56^ |
| Patient's attitude | Patient’s perceptions |  | X | 52 | 266 | ^25,55^ |
|  | Desire for children / parenthood |  | X | - | 252 | ^25,43,108^ |
| Fertility knowledge | Endorsing the benefits of banking |  | X | 52 | 1024 | ^38,55,56^ |
| REASONS FOR FOREGOING FP | | **PP** | **PP + P** | **No. of HCP** | **No. of patients** | **Ref.** |
| Clinical practice | Lack of communication with physician/No choice |  | X | - | 1024 | ^38,56^ |
| Familial & Social Support | Parents/Family/Partner disagree | X |  | 285 | 2769 | ^49,50,55^ ^22,27,43,55^ |
|  | Barriers in social environment |  | X | - | 146 | ^56^ |
| Patient's clinical characteristics | Too old |  | X | - | 482 | ^28,30^ |
|  | Too young |  | X | - | 3094 | ^22,28,56^ |
|  | Lack of time |  | X | - | 246 | ^43,44^ |
|  | Too ill to delay treatment | X | X | 209 | 361 | ^50^ ^25,44,60^ |
|  | Poor prognosis | X | X | 209 | 23 | ^50^ ^30^ |

Supplementary Table 5 continued: The reasons for pursuing or foregoing fertility preservation (FP) were sorted into 6 categories and presented as the point of view of patients and health care professionals (HCP).

| REASONS FOR PURSUING FP | | PP | PP + P | No. of HCP | No. of patients | Ref. |
| --- | --- | --- | --- | --- | --- | --- |
| Patient's attitude | Not wanting to delay treatment | X | X | 209 | 459 | ^50^ ^28^ |
|  | Wish to focus on oncological treatment (priority) |  | X |  | 230 | ^25,30,43^ |
|  | Emotional distress/Embarrassment | X | X | 9 | 1060 | ^54^ ^30,38,44^ |
|  | Fear of passing down a risk for cancer |  | X | - | 3094 | ^22,28,56^ |
|  | Fear of cancer recurrence |  | X | - | 3390 | ^22,30,38^ |
|  | Not concerned with infertility | X |  | 218 | - | ^50,54^ |
|  | Not concerned with parenthood | X |  | 209 | - | ^50^ |
|  | Not wanting biological children |  | X | - | 169 | ^30,56^ |
|  | Not wanting (more) children |  | X | - | 641 | ^28,44^ ^30^ |
| Fertility Knowledge | Poor knowledge on FP | X | X | - | 725 | ^25^ ^28^ ^55,56^ |
|  | Not knowing where to consult |  | X | - | 206 | ^27,44^ |
|  | Concerned on adverse effects |  | X | - | 110 | ^30,43^ |
|  | Expected burden of FP treatment |  | X | - | 988 | ^30,38,43^ |
| Cost | Cost | X | X | 209 | 4154 | ^50^ ^22,28,30,38,44,56^ |
